# Serum fatty acid-binding protein 4 (FABP4) concentration is associated with insulin resistance in peripheral tissues, A clinical study

**DOI:** 10.1371/journal.pone.0179737

**Published:** 2017-06-27

**Authors:** Risa Nakamura, Tsuyoshi Okura, Yohei Fujioka, Keisuke Sumi, Kazuhiko Matsuzawa, Shoichiro Izawa, Etsuko Ueta, Masahiko Kato, Shin-ichi Taniguchi, Kazuhiro Yamamoto

**Affiliations:** 1 Division of Cardiovascular Medicine, Endocrinology and Metabolism, Department of Molecular Medicine and Therapeutics, Tottori University Faculty of Medicine, Yonago, Tottori, Japan; 2 Department of Regional Medicine, Tottori University Faculty of Medicine, Yonago, Tottori, Japan; 3 School of Health Science, Tottori University Faculty of Medicine, Yonago, Tottori, Japan; University of Melbourne, AUSTRALIA

## Abstract

Type 2 diabetes mellitus (T2DM) is caused by insulin resistance and β cell dysfunction. In recent studies reported that several markers associated with insulin sensitivity in skeletal muscle, Adiponectin and other parameters, such as fatty acid-binding protein (FABP4), have been reported to regulate insulin resistance, but it remains unclear which factor mostly affects insulin resistance in T2DM. In this cross-sectional study, we evaluated the relationships between several kinds of biomarkers and insulin resistance, and insulin secretion in T2DM and healthy controls. We recruited 30 participants (12 T2DM and 18 non-diabetic healthy controls). Participants underwent a meal tolerance test during which plasma glucose, insulin and serum C-peptide immunoreactivity were measured. We performed a hyperinsulinemic-euglycemic clamp and measured the glucose-disposal rate (GDR). The fasting serum levels of adiponectin, insulin-like growth factor-1, irisin, autotaxin, FABP4 and interleukin-6 were measured by ELISA. We found a strong negative correlation between FABP4 concentration and GDR in T2DM (r = -0.657, *p =* 0.020). FABP4 also was positively correlated with insulin secretion during the meal tolerance test in T2DM (IRI (120): r = 0.604, *p* = 0.038) and was positively related to the insulinogenic index in non-DM subjects (r = 0.536, *p =* 0.022). Autotaxin was also related to GDR. However, there was no relationship with insulin secretion. We found that serum FABP4 concentration were associated with insulin resistance and secretion in T2DM. This suggests that FABP4 may play an important role in glucose homeostasis.

## Introduction

Type 2 diabetes mellitus (T2DM) is a metabolic disease that shows chronic hyperglycemia. The main pathophysiology is insulin resistance that stresses the pancreatic β-cells to augment insulin secretion and is triggered by obesity and physical inactivity [1].

Generally, obesity causes insulin resistance and creates a major risk of developing T2DM. The glucose clamp is the gold standard to evaluate the insulin-stimulated glucose uptake in skeletal muscle and adipose tissue. [2, 3]. Recent studies have reported several markers associated with insulin sensitivity in skeletal muscle [4]., including Adiponectin, insulin-like growth factor1(IGF-1), interleuin-6(IL-6), and irisin. Irisin is a cytokines and peptide, derived from skeletal muscle, known as a myokine, and exerts either autocrine, paracrine or endocrine effects Adipocyte fatty acid-protein (A-FABP), known as FABP4, and autotaxin (ATX) are secreted by adipose tissues, and have been pointed to be associated with obesity. In an earlier study, it was reported that serum concentrations of FABP4 are associate with irisin [5].

### Adiponectin

One humoral factor candidate, adiponectin, is secreted from the fat cell and improves insulin resistance. By activating AMP-activated protein kinase (AMPK) in the liver and skeletal muscle, adiponectin promotes combustion of fatty acid and accelerates glucose uptake [6]. In T2DM, adiponectin has a positive correlation with the glucose-disposal rate (GDR) [7].

### IGF-1

Low levels of IGF-1 are associated with obesity [8]. Sirbu et al. [9] reported that body mass index (BMI), a marker of total body fat, predicted low serum IGF-1 levels. In another study, serum IGF-1 levels were negatively correlated with trunk fat in obese women [10]. Thus, measurements of body composition and IGF-1 levels can predict insulin resistance. Skeletal muscle-derived IGF-1 is a potent osteogenic factor and has a role in the reinforcement of the bone by the exercise [11]. However, the direct relationship between IGF-1 and insulin resistance in skeletal muscle peripheral tissues is still unclear.

### FABP4

Adipocyte fatty acid-protein (A-FABP), known as FABP4, is one of the proteins found in mature adipocytes. It belongs to the FABP family and consists of intracellular lipid carriers that participate in regulating lipid transport and metabolism. FABP4 is secreted from adipocytes and macrophages and has recently been investigated as a marker that is closely associated with obesity and metabolic syndrome [12]. In obese mice, FABP4 deletion improved insulin sensitivity and lipid metabolic disorders [13]. Elevated serum levels of FABP4 were associated with obesity, insulin resistance, dyslipidemia and hypertension in healthy people [5]. It has been reported that FABP4 is negatively correlated with GDR in participants [14] but the relation is unknown in studies of T2DM alone. Another study targeting non-DM subjects, revealed that serum FABP concentration were negatively correlated with the mean rate of glucose infusion for the last 30 min of the clamp test used as an index of insulin sensitivity [15].

### IL-6

IL-6 may be associated with a chronic, low-grade inflammatory state, which is generally associated with obesity. However, whether IL-6 has a protective or harmful role to the condition is still controversial. Mauer et al. [16] showed that an inactivated IL-6 receptor (IL-6R) gene in mice developed insulin resistance and decreased glucose tolerance. The report showed that IL-6 may be an important regulator to activate macrophages, which are an anti-inflammatory mediator and repairs tissues in inflammatory conditions such as obesity. Additionally, IL-6 promotes the shift of glucose transporter (GLUT) 4 to a cell membrane by reinforcing the insulin signal in the skeletal muscle, activating an enzyme responsible from β-oxidation of the fatty acid and improving lipid use in the skeletal muscle, and participating in actions such as glucose uptake in skeletal muscle [17].

### Irisin

Irisin is a newly identified hormone secreted by myocytes. It mediates the beneficial effects of exercise and influences multiple metabolic pathways, such as lipid and glucose metabolism. Irisin activates the expression of peroxisome proliferator-γ coactivator-1α (PGC-1α) and uncoupling protein 1 (UCP 1) that leads to energy consumption, weight loss, and improved insulin sensitivity. Yang et al. [18] reported that irisin could recover insulin action in muscle cells by improving insulin signaling.

### ATX

An earlier study reported that ATX expression in adipose tissues was significantly upregulated in patients with insulin resistance and impaired glucose tolerance [19]. However, the relationship between serum ATX and insulin resistance remains unclear.

Given that the clamp method is complex, it is difficult to evaluate accurate insulin resistance using this method, and little is known about the relationship between those surrogate markers and insulin resistance. The purpose of the present study was to identify the major humoral factors for insulin resistance and body composition in humans. Non-DM individuals also may have insulin resistance, even if they are not obese. A surrogate marker for body fat content is BMI, which is determined by weight (kilograms) divided by height squared (square meters). A better method, however, to define obesity would be measurement of the percentage of total body fat. One of the methods to measure the percentage of total body fat is bioelectrical impedance.

We recruited individuals with T2DM and those without and measured insulin resistance and body composition markers and performed a glucose clamp and meal tolerance test (MTT).

Although it is difficult to investigate all of the cytokines, myokines, and hormones involved in insulin resistance, we measured the above mentioned factors given that these are possible insulin sensitive or resistant factors.

## Material and methods

### Subjects

#### Study population

This cross-sectional study involved 12 patients (8 men and 4 women) who were previously diagnosed with T2DM, and 18 non-diabetic control participants (10 men and 8 women). T;he T2DM patients were aged 29–67 years (mean 56±12) and the volunteer controls were aged 24–61 years (mean 35±9.3).

The diagnosis of DM was based on the criteria of the Japan Diabetes Society (JDS): fasting plasma glucose (FPG)>126 mg/dL (7.0 mmol/L) and/or 2 h-postprandial plasma glucose (PPG)>200 mg/dL (11.1 mmol/L). The participants were enrolled from 2013–2016.

#### Exclusion criteria

Patients with a history of T1DM, T2DM with medications, ischemic heart disease, heart failure, pancreatic disease, liver disease, renal failure, malignancy, inflammatory or infectious diseases, pre- or post-operation and pregnant women were excluded.

The Ethics Committee of the Faculty of Medicine of Tottori University approved the study, which we conducted in compliance with the ethical principles of the Declaration of Helsinki. We obtained informed consent from all patients and volunteers using a procedure approved by the Ethics Committee.

### Clinical assessment

All patients underwent clinical examinations before the MTT. BMI was determined as weight divided by height squared (kg/m^2^). Homeostasis model assessment of insulin resistance HOMA-IR was calculated by FPG (mmol/L) × fasting IRI (F-IRI pmol/L)/135[20]. Body composition analysis was performed using a TANITA Body Composition Analyzer BC-108 (Tanita, Tokyo, Japan). To evaluate body composition, dual-energy X-ray absorptiometry, bioelectrical impedance analysis (BIA), computer tomography (CT), and magnetic resonance imaging (MRI) may each be used. However, we used the BIA method in the present study because non-DM subjects included young females.

Body fat mass (kg), percent body fat (%), percent limbs fat (%), skeletal muscle mass (kg) and skeletal muscle index (SMI) were measured. SMI was calculated as the ratio of appendicular skeletal muscle mass/height^2^ according to the practical clinical definition and consensus diagnostic criteria for age-related sarcopenia by the European Working Group on Sarcopenia in Older People (EWGSOP) [21].

### Meal tolerance test (MTT)

The participants visited our clinic and we collected blood after overnight fasting. All participants consumed a test meal (total calories 460 kcal/1882kL, carbohydrates 56.5 g[50%], fat 18.0 g[35%], protein 18.0 g[15%]:1.6g salt) prepared by the JDS (JANEF E460F18, Kewpie Corporation, Tokyo, Japan) [22]. Plasma glucose and insulin were measured at 0 (fasting), 30, 60, 120, and 180 min following the meal. Plasma glucose was determined by the glucose oxidase method and plasma insulin using chemiluminescent immunoassays (CLIA) (human insulin CLIA kits, Kyowa Medix, Tokyo, Japan). HbA1c was measured by high-performance liquid chromatography and percentage values were converted to International Federation of Clinical Chemistry values (mmol/mol) using the HbA1c converter developed by the National Institutes of Diabetes and Digestive and Kidney Diseases [23].

#### Euglycemic–hyperinsulinemic clamp

Glucose clamp studies were performed two days after the MTT. Patients were examined in the morning after an overnight fast. An antecubital vein was cannulated to administer the infusate. A dorsal vein was cannulated and kept warm to facilitate venous sampling and provide arterialized venous blood. Using an artificial endocrine pancreas (STG 55; Nikkiso, Shizuoka, Japan), the euglycemic–hyperinsulinemic clamp was performed to determine insulin sensitivity in peripheral tissues [2]. A primed constant infusion of insulin (100 mU/m^2^/min) and computer-controlled exogenous infusion of glucose solution were used to achieve steady-state plasma insulin levels and maintain plasma glucose levels at 5.2 mmol/L (95 mg/dL). Using the insulin infusion protocol as previously reported, the steady-state plasma insulin level was 1200 pmol/L in patients with T2DM [24, 25]. The steady-state glucose infusion rate (GIR) was calculated at 90–120 min, and the mean GIR during that time was used as a marker of peripheral insulin sensitivity. The mean GIR was defined as the GDR.

### FABP4, irisin, adiponectin, autotaxin, and IL-6 assays

An enzyme-linked immunosorbent assay (ELISA) kit was used for measuring the following: A-FABP (human Adipocity FABP ELISA kit, RD191036200R, BioVendor R & D, Bruno, Czech Republic), irisin (Phoenix Pharmaceuticals, EK-067-29,Funakoshi, Tokyo, Japan), IGF-1 (IGF-1 immunoradiometric assay [IRMA], Daiichi, BPKB195, Tokyo, Japan), plasma adiponectin (human adiponectin ELISA kit, 410614, Otsuka, Tokyo, Japan), ATX (Autotaxin Sandwich ELISA, K-5600, Echelon Biosciences Inc. Salt Lake City, UT, USA), and plasma IL-6 (human IL-6 Quantikine ELISA, D6050, R & D Systems, Inc., Minneapolis, MN, USA).

### Calculation of insulin resistance and secretion indices

HOMA-IR was calculated by FPG (mmol/L) × fasting IRI (F-IRI pmol/L)/135. The insulin sensitivity index (ISI) was calculated by 10,000/√{[FPG (mmol/L) × F-IRI (pmol/L)] × [mean glucose × mean insulin during MTT]}. The insulinogenic index was measured by [insulin (pmol/L) at 30 min–insulin (pmol/L) at 0 min]/[glucose (mmol/L) at 30 min–glucose (mmol/L) at 0 min]. CPR-IR is an index of insulin resistance, calculated by 20/(fasting PG (mmol/L) × fasting CPR (nmol/L) [26].

### Statistical analysis

Statistical analysis was performed using SPSS software version 23 (SPSS, Chicago, IL, USA). Data are expressed as means ± standard error of the mean. The Student’s *t*-test was performed for the analysis of the differences between DM and non-DM groups. A *p*-value of <0.05 was considered to be statistically significant in all analyse. Univariate correlations were performed using Pearson’s rank correlation method.

## Results

### Characteristics of study participants

The baseline characteristics and chemical profiles of participants (*n* = 30) are summarized in **Table 1**. Between the two groups, mean age, BMI, AST, ALT, γ-GTP, e-GFR, TG, low-density lipoprotein (LDL)-cholesterol, FPG, 2h-PPG and HbA1c were significantly higher (*p*<0.05) in the T2DM group compared with the non-DM group.

**Table 1:** Baseline characteristics.

	non-DM (n = 12)	T2DM (n = 18)	p values
Sex (male)	10(83.3%)	8(44.4%)	0.412
Age (years)	34.8±9.7	55.2±11.6	<0.001
Height (cm)	166.3±10.1	163.7±7.8	0.463
Body weight (kg)	61.5±14.8	70.9±11.6	0.076
BMI (kg/m^2^)	22.0±3.1	26.5±4.0	0.002
TG (mmol/L)	0.9±0.4	1.6±0.8	0.002
HDL (mmol/L)	1.8±0.4	1.2±0.0	<0.001
LDL (mmol/L)	3.0±0.1	3.4±0.4	0.04
FPG (mmol/L)	4.84±0.4	6.9±0.7	<0.001
HbA1c (NGSP)(%)	5.3±0.3	7.4±0.8	<0.001
HbA1c (mmol/mol)	34±3	55±8	<0.001
Fasting-IRI (pmol/L)	46.8±29.4	75±43.2	0.039
IRI120 (pmol/L)	176.43±122.27	339.80±158.96	0.004
AUC120(glu) (mmol/L•h)	11.7±1.3	20.3±2.8	<0.001
AUC120(IRI) (pmol/L•h)	468.1±233.8	597.4±329.9	0.218
HOMA-R	1.7±12	3.9±2.5	0.003
I.I	1.4±1.2	0.9±1.2	0.271
GDR (mg/min/kg)	9.5±2.5	5.2±2.0	<0.001
IRI at the end of clamp (pmol/L)	1130.4±238.8	1280.4±330.6	0.160
FABP4 (ng/ml)	12.3±5.4	25.9±10.1	<0.001
Adiponectin (μg/ml)	7.3±4.0	4.78±2.5	0.058
hs-CRP (mg/dl)	0.03±0.04	0.22±0.20	<0.001
ATX (ng/ml)	7.6±1.7	9.3±2.0	0.016
IL-6 (pg/ml)	0.5±0.7	2.6±4.7	0.074
Irisin (ng/ml)	10.3±1.2	12.2±4.8	0.117
IGF-1 (ng/ml)	141.06±37.29	119.27±37.96	0.656

Data are means ± standard deviation. T2DM, study participants with Type 2 diabetes mellitus; Non-DM, non-diabetic study participants; *TG*, triglyceride; *HDL*-*C*, high-density lipoprotein cholesterol; *LDL*-*C*, low-density lipoprotein cholesterol; FPG, fasting plasma glucose; HbA1c, hemoglobin A1c; fasting-IRI, fasting-immunoreactive insulin, AUC, area under the curve; AUC120(glu), AUC of glucose in 120 minutes in meal tolerance test (MTT); AUC(IRI), AUC of IRI in 120 minutes; *HOMA*-*IR*, homeostasis model assessment of insulin resistance; I.I, Insulinogenic index; GDR, glucose disposal rate; hs-CRP, high-sensitivity C reactive protein; IL-6, interleukin-6; *p*<0.05 as determined by unpaired Student’s *t*-test.

In participants with T2DM, HbA1c was 7.4 ± 0.8% (55 ± 8 mmol/l, IFCC), the duration of diabetes was 4.0 ± 4.3 years and there was no progressive microangiopathy. In addition, the mean waist circumstance (WC) was 93.7 ± 12.1 cm and the mean SBP and DBP were 131.0 ± 8.5 and 81.8 ± 8.36 mmHg, respectively (data not shown).

None of the T2DM participants took oral antidiabetic drugs, though five participants used angiotensin II receptor blockers and five used statins. The fasting C-peptide and fasting IRI levels in T2DM were higher in T2DM than non-DM. Similarly, the AUC(glu) and AUC(IRI), as measured by MTT, were higher in T2DM than non-DM. AUC(glu), AUC(CPR), and AUC(IRI) were calculated by the trapezoid method in 2 h. A significant difference was found in GDR (9.5 ± 2.5 vs. 5.2 ± 2.0) and HOMA-R (1.7 ± 12 vs. 3.9 ± 2.5, *p = 0*.*003*).

We measured adiponectin, FABP4, IL-6, ATX, and irisin and found that those with T2DM had significantly higher levels of FABP4 and ATX than the non-DM group. IL-6 was not different between the T2DM and non-DM groups. One T2DM participant had remarkably high IL-6 levels, while none had inflammatory disease or other histories. Conversely, adiponectin was higher in non-DM, though not significantly.

Analysis of body composition is displayed in Table 2. Body fat mass, body fat percentage, lean body fat mass and trunk fat percentage were considerably higher in the T2DM group than the non-DM group. In females, the body composition results were higher in the non-DM group. Females of both groups have higher body fat percentages than males, especially among those with T2DM.

**Table 2:** Body composition.

	non-DM (n = 12)	T2DM (n = 18)	p values
Body fat percentage (%)	22.0±2.2	28.6±10.6	0.034
Male	22.2±5.6	23.0±3.8	
Female	24.8±5.1	40.0±10.9	
Body fat mass(kg)	13.6±5.0	20.8±9.6	0.012
Male	15.1±5.6	16.5±4.8	
Female	11.9±3.5	29.4±11.8	
Lean body mass (kg)	48.0±11.6	49.9±8.4	0.647
Skeletal muscle mass (kg)	45.4±11.1	47.1±8.0	0.653
Skeletal mass index (SMI)	16.2±2.2	17.5±1.6	0.096
Trunk fat percentage (%)	21.2±6.6	30.1±10.8	0.009
Male	20.7±7.2	24.9±4.3	
Female	21.8±6.2	40.5±13.0	

Data are means ± standard deviation. Skeletal muscle index (SMI) = skeletal muscle mass/height(m)^2^. Lean body mass = body weight–body fat mass. *p*<0.05 as determined by unpaired Student’s *t*-test.

Lean body mass, skeletal muscle mass and SMI were not different between the two groups (**Table 2**). SMI is defined as appendicular skeletal muscle mass/height^2^ according to the Report of the EWGSOP [21].

**Fig 1:**
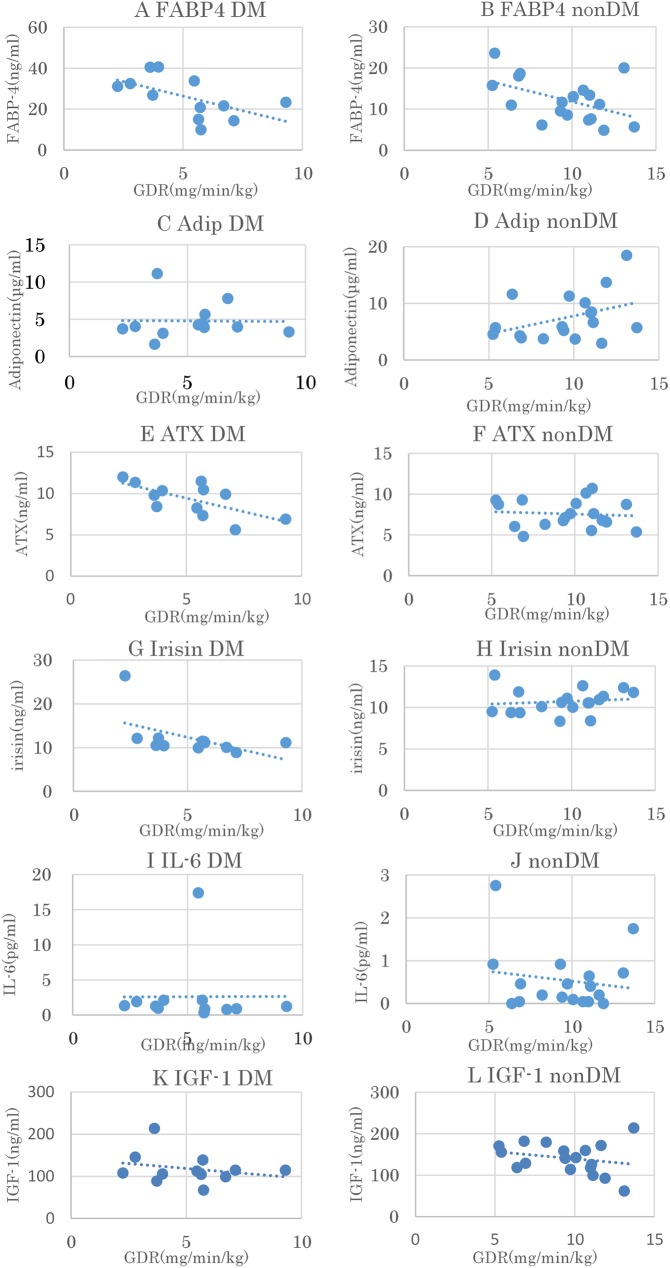
Association between several factors and glucose-disposal rate (GDR). (A) Fatty acid binding protein-4 (FABP4), Type 2 diabetes mellitus, (B) FABP4, non- diabetes mellitus, (C) Adiponectin, Type 2 diabetes mellitus (D) Adiponectin, non- diabetes mellitus, (E) Autotaxin (ATX), Type 2 diabetes mellitus, (F) ATX, non-DM, (G) Irisin, T2 diabetes mellitus, (H) Irisin, non- diabetes mellitus, (I) Interleukin (IL)-6, Type 2 diabetes mellitus, (J) IL-6, non- diabetes mellitus, (K) Insulin like growth factor (IGF)-1, Type 2 diabetes mellitus, (L) IGF-1, non- diabetes mellitus.

The plots of the relationships between the biomarkers and GDR are shown in Fig 1. We conducted a linear regression of the correlation between the GDR and biomarkers (**Table 3**). FABP4 and ATX had a strong negative correlation with GDR in those with T2DM. In the non-DM group, the correlation between FABP4 and GDR showed a weak, but positive correlation tends.

**Table 3:** Correlation with GDR.

	non-DM (n = 18)		T2DM (n = 12)	
	r	P-value	r	p values
FABP	-0.426	0.078	-0.657	0.020
ATX	-0.107	0.671	-0.601	0.039
IL-6	-0.034	0.893	-0.503	0.095
irisin	0.259	0.299	-0.591	0.056
adiponectin	0.298	0.229	0.175	0.587
IGF-1	-0.240	0.338	-0.246	0.466

Correlation coefficients were determined using Pearson’s product moment correlation coefficient test.

The association between the body composition and biomarkers is summarized in Table 4. In those with T2DM, adiponectin was negatively correlated with lean body mass and skeletal muscle mass. In non-DM participants, FABP4, IL-6 and IGF-1 were correlated with body fat mass. IL-6 in the non-DM group also had a positive correlation with lean body mass, skeletal muscle mass and skeletal mass index.

**Table 4:** Body composition and factors providing insulin resistance.

**T2DM**	FABP		Adiponectin		ATX		Il-6		irisin		IGF-1	
	r	p values	r	p values	r	p values	r	p values	r	p values	r	p values
Body fat percentage (%)	0.257	0.419	0.043	0.894	0.544	0.068	-0.34	0.279	0.507	0.093	0.154	0.651
Body fat mass(kg)	0.288	0.364	-0.143	0.657	0.549	0.065	-0.329	0.296	0.471	0.123	0.292	0.383
Lean body mass (kg)	0.02	0.952	-0.666	0.018	-0.23	0.473	0.022	0.946	-0.391	0.209	0.596	0.053
Skeletal muscle mass(kg)	0.016	0.961	-0.664	0.018	-0.236	0.461	0.024	0.941	-0.399	0.199	0.591	0.06
Skeletal mass index(SMI)	0.001	0.999	-0.798	0.022	-0.262	0.411	-0.029	0.928	-0.349	0.266	0.454	0.161
Trunk fat percentage (%)	0.25	0.434	-0.061	0.851	0.563	0.056	-0.348	0.268	0.474	0.119	0.198	0.559

**NonDM**	FABP		Adiponectin		ATX		Il-6		irisin		IGF-1	
	r	p values	r	p values	r	p values	r	p values	r	p values	r	p values
Body fat percentage (%)	0.044	0.065	-0.11	0.663	0.187	0.457	0.136	0.589	0.419	0.083	0.237	0.344
Body fat mass(kg)	0.581	0.011	-0.37	0.131	0.115	0.65	0.519	0.027*	0.404	0.096	0.484	0.042
Lean body mass (kg)	0.196	0.435	-0.451	0.061	-0.094	0.711	0.494	0.037*	-0.039	0.879	0.497	0.036
Skeletal muscle mass(kg)	0.193	0.442	-0.455	0.058	-0.093	0.713	0.493	0.038	-0.04	0.875	0.500	0.035
Skeletal mass index(SMI)	0.221	0.378	-0.488	0.040	-0.165	0.512	0.475	0.047	0.01	0.97	0.049	0.039
Trunk fat percentage (%)	0.506	0.032	-0.28	0.26	0.224	0.372	0.252	0.312	0.385	0.115	0.371	0.130

Correlation coefficients were determined using Pearson’s product moment correlation coefficient test.

*: P < 0.05.

In addition, we conducted univariate analysis between the FABP4 and several markers (Table 5).

**Table 5:** Correlation of FABP4 and clinical parameters.

	non-DM		T2DM	
	r	p	r	P
IRI(120)(pmol/L)	0.225	0.369	0.604	0.038*
Insulinogenic Index	0.536	0.022*	0.359	0.252
BMI	0.441	0.667	0.309	0.328
hs-CRP	0.255	0.306	0.314	0.320
TG	0.406	0.094	0.059	0.856
FPG	0.004	0.986	0.204	0.525
fasting CPR	0.453	0.059	0.570	0.053
fasting IRI (pmol/L)	0.435	0.071	0.441	0.151
CPR-IR	-0.263	0.293	-0.507	0.093
HOMA-R	0.419	0.084	0.449	0.143
AUC120(Glu)	0.100	0.693	0.233	0.466
AUC120(IRI)	0.331	0.180	0.508	0.092

Correlation coefficients were determined using Pearson’s product moment correlation coefficient test. CPR-IR, C-peptide immunoreactivity insulin resistance. IRI (120), IRI at 120 minutes in MTT.

FABP4 had a significant positive correlation with IRI (120) (r = 0.60. *P =* 0.038), a positive correlation tendency with fasting CPR, and a negative relationship with BMI and CPR-IR. In non-DM subjects, the insulinogenic index was positively correlated to FABP4 (r = 0.54, *p =* 0.039).

## Discussion

Our study shows that circulating FABP4 concentrations are negatively correlated with both GDR, which is a marker of insulin resistance in skeletal muscle, and serum blood insulin levels (IRI [120]) following a MTT in individuals with T2DM. Similarly, FABP4 concentration positively related with the insulinogenic index in non-DM participants.

Wu [27] reported that circulating FABP4 concentrations were correlated with glucose-stimulated insulin secretion (AUC) in healthy humans. The glucose-stimulated insulin secretion after a glucose injection also was elevated by recombinant FABP4 treatment *in vitro* and *in vivo*.

Importantly, this insulinotropic action of FABP4 is similar to the effects of GLP-1 [28], which is released by feeding. To maintain glucose homeostasis, FABP4 may stimulate the β cells and adjust insulin secretion. Additionally, FABP4 showed a positive relationship with to insulin secretion at an early stage in the non-DM group, which may be because early insulin secretion is damaged early in T2DM. Assessing this result, we assume that FABP4 enhances the process that β cells compensate insulin secretion against the insulin resistance in patients with T2DM.

In contrast, ATX correlated with GDR and body fat. However, ATX does not relate to serum insulin levels (data not shown). Earlier reports also revealed that ATX predicts insulin sensitivity (GDR) in humans [29] and mice [14]. However, few studies have researched the correlation between ATX and insulin secretion. Whether ATX reinforces insulin secretion remains unclear. However, the present results suggest that ATX is also important for insulin resistance and body composition.

We found the strongest negative correlation between FABP4 and GDR compared with the other markers connected to insulin resistance or body composition. FABP4 has been reported to have a negative correlation with GDR in T1DM, T2DM, and controls of Asian Americans [10]. We evaluated the relationship between FABP4 and several factors including GDR, body composition, and insulin secretion separately in T2DM and non-DM. As a result, we found a significant negative correlation between FABP4 and GDR in T2DM. In non-DM subjects, the relationship was similar, but not significantly. Simultaneously, we found connection between FABP4 and insulin secretion.

FABP4 is considered as an important substance concerning insulin resistance in T2DM, and for the release of compensatory insulin secretion.

In addition, it is important given that insulin resistance is a component of atherosclerosis. A previous study, reported that elevated levels of FABP4 contributed to elevation of blood pressure and an atherogenic metabolic phenotype [15]. FABP4 concentration is associated with carotid atherosclerosis [30]. FABP4 is thought to be a marker of pathophysiology and could be a target of treatment in T2DM.

FABP4 is secreted by fat cells and the blood concentration is related to obesity, diabetes, and arteriosclerosis [31]. To evaluate the exact correlation between insulin resistance and FABP4 concentrations, the glucose clamp method is required. However, this method is invasive. Accordingly, the measurement of FABP4 concentration is useful for evaluating insulin resistance in skeletal muscle if FABP4 significantly correlates to GDR, and is a predictive marker of the progression of T2DM and non-DM people [32]. The main treatment for T2DM is diet, exercise, and oral antidiabetic drug therapy. Additionally, a FABP4-specific inhibitor could become a therapeutic drug for diabetes and for arteriosclerosis, as demonstrated in mice [13].

Our study revealed no significant correlation between FABP4 and GDR in the non-DM group. This may be the result of differences in BMI in both groups. The mean BMI in the non-DM participants was 22.0 ± 3.1 kg/m^2^, and the body fat percentage was also significantly different between the two groups. We found that BMI correlated with FABP4 in T2DM but not in non-DM. According to another report, hyperinsulinemia and insulin resistance in the context of both dietary and genetic obesity improved in FABP4 deficient mice, but the effect of FABP4 on insulin sensitivity was not seen in lean mice [33]. The action of FABP4 in obesity may occur clinically, but further studies are needed to verify this point.

The other markers, adiponectin, irisin and IL-6, did not correlate with GDR. However, an earlier report showed a correlation between adiponectin and GDR in T2DM and healthy subjects combined [10]. In our study, adiponectin and GDR had a slight correlation for all participants (r = 0.45, *p =* 0.026), though no relationship was found with body composition.

FABP4 did not correlated with the HOMA-IR in the T2DM and non-DM groups in this study. HOMA-IR is a surrogate marker, which mainly reflects the insulin resistance of the liver [2]. The difference could be explained by the number of subjects or the fact that the glucose clamp method is mainly reflects the insulin resistance of muscle tissue.

The explanation of why irisin was related to the body fat percentage in T2DM and non-DM is difficult. An earlier report mentioned that peroxisome proliferator-activated receptor γ coactivator 1-α (PGC-1α) expression increased in brown fat cells and skeletal muscle in response to exercise or cold temperature stimulation [34]. In our study, we did not examine the participants in such situations. The role of irisin has been considered to be highly controversial [35]. The result from this study needs reexamination.

In the present cross-sectional study, we did not evaluate the efficacy of oral antidiabetic drugs. For antihypertensive agents, a report has indicated some influence on FABP4 that olmesartan decreases A-FABP levels [36]. It is unknown how the agent affected the results.

This study had some limitations. First, the relatively small number of participants and the differences in age and BMI between the T2DM and non-DM groups indicate that our results require confirmation with a larger study. However, the glucose clamp test is a complicated method, and it is difficult to recruit patients with poorly controlled diabetes without medications, and older non-diabetic participants with obesity. Second, to evaluate measurements of body fat mass, we could only use the BIA method because young women were included. Third, further investigation is needed to evaluate the relationships between adiponectin and other markers of insulin resistance, for example, leptin.

Leptin regulates body weight by suppressing food intake and stimulating energy expenditure [37]. Leptin also may play an important role in muscle-bone crosstalk[11].In patients with T2DM, serum leptin concentrations were positively correlated to HOMA-R, HOMA-β, and C-peptide index [38]. In another report, high-fat diet mice showed attenuated acute insulin secretion to glucose infusion, poor compensatory islet growth, and glucose intolerance [39]. Another potent insulin resistant factor is TNF-alpha. Unfortunately, we did not have enough quantity of the serum to measure leptin, TNF-alpha, and other factors in the study. Currently, we are conducting a larger study, the results of which we plan to publish in the future.

In conclusion, our study suggests indicates that FABP4 may not only be an effective marker of insulin resistance in skeletal muscle, but also enhances insulin secretion.

To our knowledge, this is the first report to compare several markers and their relationships with insulin resistance and secretion using the glucose clamp and MTT in humans. There are a large number of studies that assessed the correlation with FABP4 and insulin resistance. Accordingly, the results of our study show the possibility that, while FABP4 is important for the pathophysiology of insulin resistance, it is likely to be associated with the insulin secretion in T2DM.
