# Correction: Serum fatty acid-binding protein 4 (FABP4) concentration is associated with insulin resistance in peripheral tissues, A clinical study

**DOI:** 10.1371/journal.pone.0210932

**Published:** 2019-01-14

**Authors:** Risa Nakamura, Tsuyoshi Okura, Yohei Fujioka, Keisuke Sumi, Kazuhiko Matsuzawa, Shoichiro Izawa, Etsuko Ueta, Masahiko Kato, Shin-ichi Taniguchi, Kazuhiro Yamamoto

There is an error in the sixth sentence of the IGF-1 section of the Introduction. The correct sentence should read: However, the direct relationship between IGF-1 and insulin resistance in skeletal muscle is still unclear.

There is a typographical error in the second sentence of the Materials and methods. The correct sentence is: The T2DM patients were aged 29–67 years (mean 56±12) and the volunteer controls were aged 24–61 years (mean 35±9.3).

There was no significant difference in lean body mass between T2DM and non-DM groups. The second sentence of the fifth paragraph of the Results is therefore incorrect. The correct sentence is: Body fat mass, body fat percentage, and trunk fat percentage were considerably higher in the T2DM group than the non-DM group.

In T2DM, FABP4 had a negative correlation tendency with CPR-IR, but it was not significant. There is also no significant correlation between FABP4 and BMI. The first sentence of the final paragraph in the Results is therefore incorrect. The correct sentence is: FABP4 had a significant positive correlation with IRI (120) (r = 0.60. P = 0.038), a positive correlation tendency with fasting CPR, and negative correlation tendency with CPR-IR in the T2DM.

In T2DM, ATX had a negative correlation with GDR, and positive correlation tends with Body fat mass, Body fat percentage, and Trunk fat percentage. The first sentence of the fourth paragraph in the Discussion is therefore incorrect. The correct sentence is: In contrast, ATX showed negative correlation with GDR and positive correlation tendency with Body fat mass, Body fat percentage, and Trunk fat percentage.

In addition, the third sentence of the ninth paragraph of the Discussion should be removed. The removed sentence is: We found that BMI correlated with FABP4 in T2DM but not in non-DM.

There is an error in the penultimate paragraph of the Discussion. The paragraph should read: In conclusion, our study suggests that FABP4 may not only be an effective marker of insulin resistance in skeletal muscle, but also enhances insulin secretion.
